# Establishing a Health Equity Office: The Importance of Recentering Equity

**DOI:** 10.1089/heq.2024.0004

**Published:** 2024-08-20

**Authors:** Aswita Tan-McGrory, Amita Bey, John D. Cowden, Hans B. Kersten, Arie Nettles, W. Cody Reynolds, Valerie L. Ward, Lenny Lopez

**Affiliations:** ^1^ Massachusetts General Hospital, Disparities Solutions Center, Boston, Massachusetts, USA.; ^2^ Office of Inclusion and Health Equity, Monroe Carell Jr. Children’s Hospital at Vanderbilt, Nashville, Tennessee, USA.; ^3^ Office of Equity and Diversity & Department of Pediatrics, Children’s Mercy Kansas City, Kansas, Missouri, USA.; ^4^ Department of Pediatrics, College of Medicine, Drexel University, Philadelphia, Pennsylvania, USA.; ^5^ Division of Ambulatory Pediatrics, St Christopher’s Hospital for Children, Philadelphia, Pennsylvania, USA.; ^6^ Office of Health Equity and Inclusion, Boston Children’s Hospital, Boston, Massachusetts, USA.; ^7^ Sandra L. Fenwick Institute for Pediatric Health Equity and Inclusion, Boston Children’s Hospital, Boston, Massachusetts, USA.; ^8^ Department of Radiology, Boston Children’s Hospital, Boston, Massachusetts, USA.; ^9^ Harvard Medical School, Boston, Massachusetts, USA.; ^10^ Department of Medicine, University of California, San Francisco and the San Francisco VA Medical Center, California, USA.

**Keywords:** disparities, equity, health equity office

## Abstract

**Objectives:**

The Pediatric Health Equity Collaborative (PHEC) set out to describe the best practices for establishing a health equity-focused office within a clinical setting.

**Study Design:**

Survey and in-depth interviews of the members of the PHEC comprised pediatric care delivery systems in the United States and Canada.

**Methods:**

Human-centered design methods were utilized in an iterative fashion to develop and agree on survey and interview domains. The final seven domains were as follows: (1) history of the office, (2) general description of the office, (3) position of the office in the organization, (4) budget and finance, (5) stakeholders, (6) community engagement, and (7) measuring outcomes. Interviews were analyzed using an applied thematic approach to inductively identify themes until saturation was achieved.

**Results:**

PHEC participants articulated several key implementation factors in the development of a health equity office. First, the history of the office is important and has the potential to determine the office’s scope of work and sphere of influence. Second, a health equity office can provide crosscutting organizational direction, stability, and execution of equity efforts, reducing the effects of siloing. Third, high-level leadership buy-in provides time and financial resources. Finally, a health equity office should be centrally involved in the collection, analysis, and reporting of equity-focused metrics.

**Conclusions:**

A health equity-focused office can play an integral and sustaining role in representing and focusing equity efforts across an organization, measuring processes and outcomes, and helping to develop the equity mission and vision.

## Introduction

The arc of disparities and equity in health care is marked by three seminal events: the 2003 Institute of Medicine report *Unequal Treatment*, the COVID-19 pandemic, and the murder of Mr. George Floyd in May 2020. *Unequal Treatment* provided the body of evidence and data showing that racial and ethnic disparities in health care existed. The COVID-19 pandemic revealed the impact of the social and political determinants of health on mitigating and addressing COVID-19 and the disparities it created. Mr. George Floyd’s death soon thereafter was the accelerant for a nationwide reckoning on race and policing and the existence and prevalence of structural racism throughout our society. In the wake of all of these, there is an increasing urgency for health care organizations to implement an equity strategy, reflected in new attention paid to equity by governmental, accreditation, patient safety, and trade organizations.^1–3^ Health equity organizational efforts and initiatives require significant resource investment and planning in order to effect organizational structural and cultural changes. Culture change occurs once a new way of operating takes hold in an organization through a long-term iterative process, similar to what is described in the Kotter Model about organizational change.^4^

Little has been published on how to organize and sustain long-term health equity efforts in health care delivery organizations. Most prior research has focused on personnel diversity and inclusion hiring and training efforts and fairness in compensation and promotion, but there has been less emphasis on equity.^5–10^ Organizationally, there has been growing interest in having C-suite level/executive Diversity, Equity and Inclusion (DEI) leadership positions. According to LinkedIn data, the number of people globally with the title “Head of Diversity” more than doubled (107% growth) over the last 5 years. The number with the title “Director of Diversity” grew 75% and “chief diversity officer” 68%.^11^ In qualitative interviews with 40 Chief Diversity Officers (CDOs), it was noted after the summer of 2020, the roles of CDOs became vitally important to organizations.^9^ This increased interest of having this position in the C-suite level is indicative of the need for a corresponding health equity office to execute on the work.

While there are multiple frameworks for achieving equity in clinical care/quality, or diversity and inclusion, there are fewer frameworks addressing these jointly.^5,8,12–19^ Unlike other offices within a health care setting (e.g., quality and safety), the success of a health equity office is heavily influenced by the prevailing focus of the organization’s culture, leadership, and funding, as well as the larger national discussions on topics such as structural racism, bias, disparities, and equity. Prior to the onset of the COVID-19 pandemic, the Pediatric Health Equity Collaborative (PHEC) set out to describe the best practices for the establishment of a health equity-focused office within a clinical setting specifically within pediatric hospitals. Although there is emerging research on the need for and importance of equity efforts and initiatives in hospitals, there have been few peer reviewed studies on how to start, establish, and grow an equity office.^20–22^ We surveyed and interviewed the members of the PHEC comprising clinical, administrative, and operational leaders from eight pediatric health care delivery organizations in the United States and Canada, to better understand the key elements that are needed for a successful health equity office launch.

## Methods

### Pediatric Health Equity Collaborative

In 2013, PHEC was formed and consisted of 16 research and clinical professional experts working in 10 pediatric care delivery systems in the United States and Canada (8 pediatric and 2 pediatric/adult hospitals), which has now expanded to 21 members representing 11 organizations. PHEC members included physicians, nurses, social workers, human resource specialists, and administrative staff.

### Multidisciplinary Participatory Approach

#### Generation of research question

PHEC members met in person in 2018 at Nationwide Children’s Hospital in Columbus, Ohio, to discuss and arrive at a consensus about a research question relevant to the intersection of equity and Pediatrics (a complete list of participants is provided in the Supplementary Appendix). Led by an expert facilitator, human-centered design (HCD) methods were utilized in an iterative fashion to optimize participation, collaboration, consensus, and priorities. HCD is a process with the purpose to elicit needs, desires, and experiences of people to further define a problem and develop consensus and solutions.^23–25^ Once consensus was reached to describe the best practices for the establishment of a health equity-focused office within a clinical setting, an in-depth literature review was conducted including peer-reviewed publications and the gray literature. This literature review was updated in 2023.

#### Consensus development of study domains and data-gathering approach

Several remote meetings were conducted to arrive at an appropriate participatory methodological approach. Given the lack of prior empirical evidence, PHEC members decided to focus on describing the experience of each PHEC institution in designing and implementing a health equity office. HCD techniques were used again to generate the domains that would be of most value. In an iterative fashion, broad categories were narrowed, and consensus was reached on key themes. The group selected 7 final domains for the survey, which consisted of 49 questions. The 7 domains were (1) history of the office, (2) general description of the office, (3) position of the office in the organization, (4) budget and finance, (5) stakeholders, (6) community engagement, and (7) measuring outcomes.

Given the large number of domains, a two-step data gathering approach was utilized. First, domains that were primarily background information on an institution were included in a survey that was completed by each PHEC member about their institution. Second, the 7 survey domains were explored through in-depth semi-structured interviews with an interview guide consisting of 15 questions. An in-person meeting was held to allow for PHEC members to meet and interview each other in pairs. Prior to the interviews, PHEC members received the results of the informational survey for the institutional representative they were interviewing to allow for more in-depth questioning. Interviewers were encouraged to ask additional probing questions in addition to the interview guide. Interviews were recorded and transcribed verbatim by professional transcriptionists. Complete survey and interview questions are available in the Supplementary Appendix.

### Qualitative Analyses

A remote meeting was held to review best practices for qualitative content analysis of the interview transcripts.^24–26^ Interviews were recorded and sent out to be transcribed by professional transcription services. Each PHEC member independently analyzed the transcript of the interview they conducted using an applied thematic approach to inductively identify themes. In addition, two study authors (L.L. and A.T.-M.) independently analyzed and identified themes for all of the interviews. Identified themes were compiled and organized by interview question into tables (W.C.R.). These were reviewed and discussed for consensus by PHEC members both individually and in a remote meeting. All participating members agreed that thematic saturation had been achieved in reviewing the final key themes of the study (Tables 1 and 2).

**Table 1.** Hospital Characteristics

Hospitals	Start date	Who started the office	Titles of personnel	Leader of the office	Scope of work	Where the office sits in the organization chart	Who does the office report to	Are there partnership(s) with other stakeholders in the organization? Which?
St. Christopher’s Hospital for Children	May 14, 2019 We do not have an official pediatric health equity office but started with a hospital-wide Health Equity Committee.	Physician leader and Professor of Pediatrics. Previously, the Associate Chair, Community Engagement and Health Disparities.	Multidisciplinary team of engaged providers and staff.	Dr. Hans Kersten	Community engagement, screening, and intervention on the social determinants of health (SDOH), education.	Not in organization chart	Senior Leadership and CMO	Physician, nursing, and staff leaders in health equity. Senior leadership, CMO, CNO, Director of HR.
Children’s Mercy Kanas City	Strategic Planning in 2008. Office in 2009.	Chief Operating Officer, John Cowden, MD—Medical Director, Gaby Flores	Chief Diversity Officer, Medical Director, Administrative Director, Health Equity Integration Project leader, Project Manager, Office Manager, Health Literacy Coordinator, Education Coordinator, Language and Culture Coach (—three to four Summer Interns)	Co-leaders: Chief Diversity Officer, Medical Director, Admin Director	Cultural Competency Education programming for staff; Employee Resource Groups—Administration and support to groups; Qualified Bilingual Staff program administration and oversight; Equity and Diversity Council and five work groups; Health Literacy Committee Consejo de Familias Hispanas (Hispanic Family Advisory Board); Faculty-Trainee Diversity & Inclusion Committee; Health Equity Integration Project/ Health Equity Surveillance; Community Engagement programs	Under CDO with a dotted line to clinical effectiveness (Quality and Safety division)	CDO up to COO	Hospital Board of Directors; internal leaders; E&D Council serves as ambassadors across the institution; UMKC Schools of Health Professions KC Chamber of Commerce; D&I Consortium in region Community Partners, and many others
Nemours/Alfred I. duPont	May 12, 2002	Drs. Kevin Churchwell and Kirk Dabney	Medical Director; Principal Research Scientist; Health Equity Research Associate; Research Scientists/Data Analysts (two part-time); Administrative Director; Health Equity Program Coordinator (one open position for Diversity and Inclusion Consultant)	Dr. Kirk Dabney	Quality and Value; Research; Cultural Competence; Language and Health Literacy; Community Engagement; Workforce Diversity and Inclusion	Under COO	Dotted lines to current divisional CEO and directors of Marketing/Communication and Community Engagement	NHEC (Nemours Health Equity Collaborative) formed in 2015 includes stakeholders from all levels of education, local government, for profit and not-for profit, Nemours leadership
Monroe Carell Jr. Children’s Hospital at Vanderbilt	The Office of Inclusion and Health Equity (OIHE) started in December 2011	Developed by the Department of Pediatrics and hospital senior leadership who provided funds for executive leadership training with the DLP to develop the office.	One Director and one Associate Director	Dr. Arie L. Nettles, Founding Director	**Initiative 1: Education and training—**Comprehensive training curriculum, introductory workshops: Blind spots and unconscious bias—”No, Not Me!,” Cultural Competency Modules, Unconscious Bias Training: Cook-Ross Program, Unconscious bias “booster shot,” Specialized Group Training, Respect at Work: Eliminating Sexual Harassment, Respect at Work: Ongoing series, Consultative services, Specialized strategic planning sessions, Online self-paced modules via Percipio, Student intern program **Initiative 2: Ongoing evaluation and improvement—**Evaluation of patient satisfaction based on race/ethnicity, language, and insurance status, pre-and post-test evaluations to assess individuals change in knowledge following UCB training **Initiative 3: Strategic planning and sustainability**—Expansion from pediatric hospital to entire medical center enterprise, ongoing collaboration with community partners, VUMC end-of-Life committee, and VCH Morbidity, Mortality, and Improvement Conference consultant **Initiative 4: Research and evaluation—**Pilot study to evaluate health care providers’ knowledge, attitudes, and beliefs about cultural competency, pilot study to evaluate health care providers’ knowledge, attitudes, and beliefs about cultural competency, studied physician perceptions of cultural competency training	In the Monroe Carell Jr. Children’s Hospital at Vanderbilt Department of Administration	Reports to hospital C-suite	Monroe Carell Jr. Children’s Hospital at Vanderbilt Department of Administration; Vanderbilt University Medical Center (VUMC) Department of Pediatrics; VUMC Learning and Development, Human Resources; VUMC Employee and Labor Relations, Vanderbilt University School of Medicine; LGBTI Health, Vanderbilt University School of Medicine, Vanderbilt Health
Boston Children’s Hospital	The Boston Children’s Hospital Office of Health Equity and Inclusion started on October 1, 2017	Dr. Kevin B. Churchwell, Boston Children’s President and CEO	The office began with a Medical Director (subsequently promoted to a Senior Vice President), Administrative Director, Program Coordinator, and part-time research associate (Fellow/Instructor level physician). The office will be expanded to include other part-time clinicians as senior faculty advisors, a statistician, a senior administrative associate, and a project manager. Also, the Administrative Director role was elevated to a Program Director role. The office has liaisons from each hospital departmental/division to align the work with the office and the hospital’s Declaration on Equity, Diversity and Inclusivity	Valerie L. Ward, MD, MPH, Senior Vice President and Chief Equity and Inclusion Officer	The office has four main areas of focus: research into health care disparities, education in the delivery of culturally effective pediatric health care for all faculty, trainees, and staff, workforce diversity (includes pathway programs, support for trainees/fellows, and faculty recruitment, development, retention and promotion), and patient navigation/health care literacy. Principles of quality improvement and patient/family/employee experience are embedded within these programs. In collaboration with the Program for Patient Safety and Quality at our hospital, health equity safety and quality metrics are developed, monitored, and reported out to the Board of Trustees on a regular basis	The office sits within the Department of Health Affairs and the Senior Vice President/Chief Equity and Inclusion Officer	Directly to both the President/CEO and to the Executive Vice President of Health Affairs	The office has multiple internal and external stakeholders. These include hospital Senior Leadership, Harvard Medical School, other Harvard-affiliated hospitals of the medical school, national organizations such as PHEC, Solutions for Patient Safety, Children’s Hospital Association; and all hospital staff and faculty
The Hospital for Sick Children	The Center for Innovation & Excellence in Child and Family-Centered Care was established in 2011	The hospital President/CEO and the CNO	30 FTEs in the Center. We have a Senior Manager, who, in addition to managing a number of operating areas, also oversees health equity initiatives. She is a social worker and educator by background. We also have a Health Equity and Patient Experience Coordinator. She is a social worker by background.	Director, RN, MBA	The Center includes a number of operating areas (e.g., Interpreter Services, Family Legal Health Program, The Office of Patient and Family Experience, Spiritual and Religious Care, Family Center, and Family Spaces) as well as programmatic areas including Patient and Family Engagement and Health Equity. Health equity-related initiatives have included the collection of sociodemographic data from patients and families, enhancing accessibility for people with disabilities, developing and delivering education focusing on cultural competence, developing an Indigenous Health Strategy, etc.	The Center is situated within the Learning Institute of the hospital	Vice President, Learning Institute	Internal stakeholders
Nationwide Children’s Hospital	The Office of Health Equity was created in September 2013	Dr. Olivia Thomas, Ambulatory Division Chief	Chief of Diversity and Health Equity (0.5) Medical Lead (0.3) Director (1.0) Researcher (0.5) Data Analyst (0.5) Administrative Assistant III (0.5)	Dr. Olivia Thomas, Chief of Diversity & Health Equity, Dr. Linda Stoverock, Chief Nursing Officer, Rick Miller, President and COO	Health Equity Across the Organization: Office of Health Equity: Quality Improvement: adding REL categories to all data collection tools; Health Equity Inventory: database capturing health disparity projects; Health Equity Snapshot: quarterly distribution, highlighting equity activities; EPIC: language services, SDOH screening tool, sexual orientation, and gender identity; Medical Legal Partnership Community Wellness: Healthy Neighborhoods Healthy Families: focus on housing (e.g., Healthy Homes), education (e.g., mentoring, Upward Bound), health and wellness (e.g., school-based health, mobile care center), safe neighborhoods (e.g., Block Watch), and workforce development (e.g., Career Gateway); Infant mortality: Ohio Better Birth Outcomes Center for Population Health & Equity Research: Medicaid health outcomes and policy research Community wellness and housing research health equity focus populations quality improvement projects and research; Annual NCH health equity research conference	Office sits in the Chief Nursing Office	Reports directly to the Chief Nursing Officer and COO	Internal: Members of the Data Enhancement Team, Health Equity Collaborative, and the Sexual Orientation & Gender Identity Subcommittee External: PHEC, and The Ohio State University: Department of Pediatrics, Medical School, Office of Diversity & Inclusion, and the Kirwan Institute for the Study of Race & Ethnicity
Children’s Health	We do not have an Office of Health Equity.	Rick Miller, President & Chief Operating Officer	Health Equity Manager, Scientist, Specialist. Director of D&I, Program Manager D&I	Director of D&I, Workforce Development and VP, Chief Health Equity, and Inclusion Officer for the health system	Health equity efforts concerning REaL data, Standardized Screening for SDOH, Disparities Research, Alignment with Quality & Patient Safety. D&I efforts also involve HR such as equitable hiring practices and talent management, employee satisfaction, retention, and education, stratification of the talent pipeline, career mobility, and apprenticeships	Sits under the Senior Vice President of Strategy, Innovation and Network Development Officer	Report to the Senior Vice President of Strategy, Innovation and Network Development, who, in turn, reports to the President of Children’s Health Care Network and Chief Strategy Officer. The Director of D&I/Workforce Development has a dotted line reporting relationship to the VP, Chief Health Equity & Inclusion Officer and a solid line to the Senior Vice President of HR/Chief Human Resources Officer	The following departments: Quality & Patient Safety, Patient Education & Engagement (they also have Health Literacy in their purview), Department of Support Services (Language Access Services, Social Work and Pastoral Care), Business Analytics and Data Intelligence, Marketing, Nursing leadership, UT Southwestern (faculty), UT School of Public Health (UTSPH), and the Children’s Health Center for Population Health Research

CNO, Chief Nursing Officer; FTE, Full Time Equivalent; REL, Race, Ethnicity, Language; CMO, Chief Medical Officer; UMKC, University of Missouri - Kansas City; CDO, Chief Diversity Officer; UCB, unconscious bias; VCH, Vanderbilt Children’s Hospital.

**Table 2.** Results of Interviews with Quotes

Themes	Illustrative quotes
1. Importance of having an office	
Advantages	
Demonstrates importance of the work	“We need people to buy into the health equity, diversity, and inclusion importance. And around here, they definitely need the evidence of, well, where are things not as equitable as they could be? Where are the disparities? What has our own data and our own research shown for our population of patients and our diversity of patients? Where can we improve? We have found—because when we did cultural composite training about five or seven years ago in our hospital, our healthcare providers, our physicians and surgeons especially, definitely listened and participated once they heard that the stories that we were talking about were our own stories, in areas where we could improve, to deliver more effective pediatric care to patients and families. So we know, from that experience, that we have to show our own internal data, and how we have gaps in the data, or how we can close the gaps. That’ll be our way to get buy-in and more stakeholders.”
Shows organizational commitment	“The other piece is that when you have an institutional commitment to this area and this work, when you submit grants, it is very advantageous because there is a full curriculum that shows how your division has been actively involved. And so that really has been a plus—Because it’s being asked on the grant applications now all the time. So they can put in a robust answer to show, ‘Yes. Our organization is committed to this.’ Which strengthens their grant proposal.”
Allows for focused development of strategic plan	“I think the thing that has helped us most has been knowing that—looking at equity, diversity, and culture, all of these were a part of the hospital’s strategic plan. So that new plan, I think, started in 2017, and it was a five-year plan. So not only do we have just our own department looking at this, but we know the whole hospital knows that there is a need to focus in on equity. So I think that’s made this easier for us. But I don’t think we’ve had to force anybody to do anything with this.”
Coordination of scattered DEI efforts across organization—prevents siloed effort	“[T]here are people who are doing work that would be considered health equity, but as you said, there’s no one whose title is Director of Health Equity. And the kind of the outside player about that is that we are a free-standing children’s hospital, but we’re affiliated with a university who’s doing some work on health equity, and we have not been able to kind of mobilize or collaborate with them in any way. And so although I think we need to do it on our own, I think we also need to think about are there ways to partner with our kind of academic institutions to kind of work with them more deliberately.”
Helps establish funding	“Our funding comes from the operating budget of the office. We submit our budget requests just like any other office. We have been very fortunate to have received additional funding for some innovative initiatives, including our medical staff organization has funding, this year, an inaugural pediatric health equity grant, and then they’ve also decided that this will be a regularly recurring grant in pediatric health equity and inclusion. And just a real delight because that grant will be administered out of our office, but it’s their funding. So that’s, again, buy-in and traction within the organization saying, ‘We like the work you’re doing. We want to help to promote health equity scholarship.”
Dedicates personnel	“So what we’re doing is we’re actually growing the office. We’re making it clear that we have more and more things being asked of us as an office, and that we need more staff and faculty. And it’s being taken seriously. And so we are adding staff.”
Facilitates collaboration across organizations	· “I think that, at our organization and someone who does not have a health equity office, that there are pillars of people doing passionate work, but the problem is that it’s not structured and organized, in a way. And so sometimes you’ll learn about … someone doing similar work in another section, so you feel like you’re doing good work and you’re getting recognized, and, at the same time, someone doesn’t know that you’re doing that. But there are other pillars of strength around … other things that people have worked very hard to establish at our organization, so it feels like there’s a lot of great work going on, but it’s disparate in the sense that there is not an overlying sense of how we’re going to do this.”
Facilitates multilevel programming across organizations	“I think bringing in experts and leveraging our connections through PHEC, the DLP, so that staff and faculty here can see what’s happening across the country. And I think sometimes, hearing what’s happening nationally can help get buy-in from other people to feel like we want to be on that national stage as well and engaging in best practices. And I think partnering with—strategically partnering with certain offices, and aligning it to their own priorities or needs can help get buy-in.”
Sustains momentum	“Well, as a new office, we’ve had so much momentum. It’s just been almost like a—it’s been phenomenal. There’s so much excitement around this new office. People are reaching out to us. They want to collaborate with us.”
Disadvantages	
Creates scope creep	“What we’re doing is we’re actually growing the [DEI] office. We’re making it clear that we have more and more things being asked of us as an office, and that we need more staff and faculty.”
Many stakeholders and competing demands	“Before you know it, your small core group of 5 has become 55 different key stakeholders who have a piece of that pie.”
2. History of where the office starts matters	
Advantages	
Important to have a dedicated office	“With our office, we have the opportunity to blend equity, diversity, and inclusion because they have to be intertwined, we know that. We are very much heavy on the health equity scholarship side of it, and inclusion—even inclusion scholarship and programming, and intentional efforts.”
Helps to focus and organization efforts	“[O]ur office was established in response to a charge by senior leadership at the children’s hospital, in the Department of Pediatrics, to create a centralized infrastructure that would be able to meet the education and training needs around the cultural competency, cultural awareness, and cultural sensitivity for our workforce. So this mandate was made in response to the idea or the realization that our patient population was becoming increasingly diverse … So there was a need to make sure that our faculty and staff were able to provide culturally sensitive and aware care in simple to elevate the quality of care that the patients and patients’ families received.”
Make equity the focus not just in HR	“I really do not think that D&I and equity should be separated. So in some organizations—right?—D&I is kind of tied at the hip with HR. And others, the quality equity efforts are kind of tied at the hip in quality and safety. And so they’re so closely related that I think doing that misses a picture. So I have resisted several times when senior leaders have said, ‘Well, shouldn’t we sort of separate them? There’s a lot going on in each one of them,’ which I agree, but I think if we separate them we’re going to really miss out on how they’re tied together.”
Disadvantages	
Where in the organizational structure you start determines the scope of influence	“So I think the main thing that I try to do is connect it to things that really matter to our senior leaders. And that would be tying it to things like safety, which is a huge strategic priority, also to quality, another big one. We’ve also tied it a lot to the consumer experience. Consumerism, right now, in our healthcare organization is very big. And then, more importantly, tying over the risk and risk management, so just trying to hit those big, key points that, I think, matter to an organization and connecting the dots for senior leaders and for middle managers and everywhere in-between to make sure that they understand what health equity is and how it’s meaningful to their role.”
Funding limitations	“It [the budget] is tiny. I have put the majority of the funds for the team in things like education, travel because I really feel like we’re in the learning—a lot of learning needs to be had. And so prioritizing educational conferences and things of that nature has been important to me. The other thing that I put budgets in there for is for—not consulting but what we call professional services.”
3. Importance of high-level and C-suite location	
Advantages	
Sends strong message of importance of DEI	“I think that, to be really honest, if I were to report in to quality, I think we would be much more diluted and would not have the same support that we do right now. So I think that getting as close as you can to the CEO is important. But I think it’s also about lining up where there is the support that you need for your initiatives.”
Allows high-level title	· “[T]hat’s what people recognize, and they understand what that role [Chief Diversity Officer] does. And so I think that there’s importance in a name and people have to identify with that name and understand and respect that role and what the person is doing in that role. And that person has to be empowered and part of that empowerment is the name that you give the individual.”
Creates large scope of influence	· “I think where I’d like to see it in a year from now, I think we need to have somebody named as Chief Diversity Officer. We need to have this be an enterprise-wide office and not just for one part of the organization. I think we would be influencing across the board culture change, but down the road also what the employment structure looks like in the organization so that we would have providers, in particular, that are more of a mirror image of our patient population so we will have that kind of influence.”
More opportunities for funding	“[W]hen it comes to getting funding or maybe even getting support for an initiative that you’re trying to push forward, if you have somebody advocating for that work at the senior table, then there’s a better chance of getting buy-in and getting executive support so that you can actually run the initiative.”
Helps with succession planning for leadership	“[T]he departure of our chief operating officer who was a real diversity champion since the very beginning left the institution back in August. And so we lobbied to have the chief diversity officer role be created even if it was just adjunct, not a full 1.0 dedicated FTE, but someone at the table because she was no longer going to be our superhero defending our cause at that table”
Facilitates communication and planning	· “[W]e didn’t have this where we reported directly to the CEO. And I think that this has to directly report to the CEO or else it doesn’t work very well because you have to go through multiple layers and the message gets twisted and turned by the time it gets to the CEO.”
Facilitates messaging of DEI across organizations	· “I knew that our office was getting buy-in through an organization when an orthopedic surgery reached out to us. And I call it an equity consult. They asked us, “Would you help us recruit more diverse patients into a multi-center study that we have going on nationally?” We were like, “Wow, that’s an equity consult stat.” They see the value of our office, and that we have something that we can bring to their project.”
Helps organization-wide rollout of programming and training	· “Our CEO dedicated our entire assembly to our training that was going to roll out, and he gave a lecture about why this was necessary, gave some examples across the medical center as to why we need to be engaging in this work, and why this work is important in our space. And then we had a breakout. There were a couple hundred leaders across the medical center that participated in that. Then we had a breakout session that was attended by about 200 leaders, where we were able to give them a heads-up of what the curriculum would look like, and who was going to be trained, and how it was going to be rolled out across the medical center, so it wasn’t just thrown on them. They had an idea of what was coming down the pipeline.”
Enables for partnering with development and fundraising	· “I do think that the org structure that we’re in right now as a team is excellent. It falls under strategy and innovation, which sometimes feels a little odd because we do so much in terms of quality work, just tying to quality. One would think it would fit more squarely in that. But because we are doing disparities work, it’s something innovative. We’re also researching. We’re looking at implementation science to mitigate disparities and things of that nature. We wound up in this other shop, which has been a wonderful place to be. In particular, right now, because of where we are as an organization, the team in network and innovation does receive a lot of attention. They’ve received a lot of support, tons of support, from the president of network and innovation. And he is tied to the CEO. So I have a very easy way to get back to a strategic alignment.”
Disadvantages	
Vulnerable to changes in CEO, C-suite, and senior executive leadership	“I think when we think about creating a vision, I think that the coalition that we’ve built so far, through the Disparities Leadership Program (DLP) group, and then other allies like the SVP of HR and the chief quality and safety officer, have allowed us to try and create a vision for health equity that, ideally, will be really institutionalized. And so I think we’re spending a lot of time creating that shared vision so that if there are other changes, as there are other changes to hospital leadership, that this is a—we have a broad enough coalition that we’ve assembled, that this vision will be—we’re figuring out how do we roll up this vision with some of the other strategic priorities for the hospital around patient and employee experience.”
Large and complex organizational structure may hinder mission and implementation at the local level, and it may also provide for more opportunities to address the needed DEI work with a multipronged approach	“Our Office of Health Equity and Inclusion came into fruition at this hospital; however, we already have an Office of Community Health. And it’s interesting because a wonderful pediatrician is the executive director of the Office of Community Health, and we have a lot of community engagement. When our Office of Health Equity and Inclusion came into fruition, it was more centered on what’s going on inside the hospital. Not to say we’re not going to engage with anyone who gets discharged and becomes an outpatient. We have ideas for projects that span the inpatient realm to the outpatient. But our work is more within the enterprise. We could always expand but we do have an office that is already focused on community health.”
Competing organizational demands	“That’s the tricky piece is to make sure that as things change, a new CEO arrives, the contracting and the way health care as a business that shifts around. Politics and policy changes that we continue to come back to this touchstone and say health equity is not a new idea or a new thing for us to worry about. It’s always been the thing that we’re worried about and we cannot lose sight of that. We have to keep calling it out explicitly as things might threaten it. Either on purpose or not on purpose. Right? And so to me it’s hopeful that as we move forward into uncertain times the hospital as a whole already has this spirit about it. We’re not going to have to be a small group trying to get attention for this issue.”
4. Integration into organization mission and strategic plan	
Advantages	
Alignment with institutional priorities	“Our strategic directions … make diversity and inclusion intentionally part of our organizational thread. This image [strategic directions logo], it’s very visible. This goes out in all of our newsletters. When you walk into the hospital, you see it, and it’s something that is known. Everybody at our institution knows that these are our four strategic directions.”
Immediate senior leadership buy-in	“I think the most critical thing that we did, that was helpful in terms of establishing buy-in, is we started from a top-down approach. For all of our pieces within our curriculum or our training, we started with leadership. Senior leadership were the first people to go through each of these modules, and then, from there, we started with staff leaders, and then house staff, after that. So the training, it’s not mandatory, but it is highly recommended or highly suggested. And by having senior leadership go through it initially, it wasn’t hard to trickle down.”
Alignment with organizational “currency” (i.e., research, education)	“[W]e have a very large institution, so there’s a whole research institute. And so having research centers for disparities and diversity is helpful, and then we just created the population health that’s also helpful … At lots of different levels, we have things going on. So even though those don’t all fall under the office of health equity, they help continue our momentum. We are working together. So we collaborate.”
Allows responsiveness to shift in external pressures on organization	“We heard over and over that [our hospital] does not have an Office of Diversity, or equity, or inclusion, or a multicultural affairs office, but the other five major teaching hospitals do. So that’s somewhat peer group pressure, or within your own system. And why don’t we have one? We heard it from our residents that when medical students come to our table at recruitment meetings … they hear that we don’t have an office, so they wonder whether or not there’s a real commitment to have them coming to your hospital. So it came up over and over … I think that that can create some urgency as well for us to demonstrate how we are addressing it. And how can [our hospital], even within a city that has a kind of negative reputation when it comes to race and racism, be a champion for equity.”
Creates funding opportunities	“I think because our office has been able to demonstrate robust programming and defuse coalitions across the hospital, we recently, for this next budget season, will be increasing its existing hospital funding that’s shifting over and being administered by our office for programs that fit with health equity around training recruitment and some educational programming related to culturally effective pediatric healthcare, that was previously administered by HR. And HR is still holding funding for some of the more diversity programming, but some of that funding was re-appropriated to our office because it was more fitting for us to do it”
Priority of office and mission highlighted	“I think that it would’ve been helpful for me to take deliberate steps in actually setting up an office of health equity and inclusion instead of it being folded under the broader mandate of the Centre for Innovation and Excellence. I think that if we had carved out an actual office with a very specific plan in terms of goals and objectives that we probably could’ve been able to further the work.”
Disadvantages	
Competing priorities leading to diffusion of focus	“I think what the question is trying to ask is whether this value seen in the notion of health equity and inclusion in the organization—and I would say that—I would say that pockets of the hospital see it as being really important work. I would not say that it is organizationally understood or that it’s our organizational culture to necessarily be supportive of equity. It’s not to say people are not supportive, but I wouldn’t say that it’s the culture of the organization.”
5. Additional key elements for planning the office	
Making equity the system focus, not just the responsibility of HR	“I have found that partnering with HR, specifically through the executive learning area, learning and development, they’ve been key for many reasons. They had the neck in there to really track and measure and distribute and develop … and so that gives you an idea of where the trainings are and a lot of times you can pretty much map and identify those areas that may be struggling a little more that have opportunity. Now, we’ve not made anything mandatory. However, we’re partnering with Human Resources part of their own—but they have adopted as part of their onboarding is our training now for new hires. It becomes automatic for them. But you don’t have to create something separate. They’ve already got that machinery, you just help advise on what’s going to be in there, how to do it. And all the folks have been trained by us and all my team members with it”
Need for runway time to develop mission/vision, develop stakeholder buy-in and engagement	“We need people to buy into the health equity, diversity, and inclusion importance. And around here, they definitely need the evidence of, well, where are things not as equitable as they could be? Where are the disparities? What has our own data and our own research shown for our population of patients and our diversity of patients? Where can we improve? We have found—because when we did cultural composite training about five or seven years ago in our hospital, our healthcare providers, our physicians and surgeons especially, definitely listened and participated once they heard that the stories that we were talking about were our own stories, in areas where we could improve, to deliver more effective pediatric care to patients and families. So we know, from that experience, that we have to show our own internal data, and how we have gaps in the data, or how we can close the gaps. That’ll be our way to get buy-in and more stakeholders.”
Need clinicians, administrative and implementation staff, data analyst, and IS	“[Y]ou need to have a blend of a lot of skill sets on your team … you need to have people that have a clinical background … you need to have either nurses or advanced practice nurses, PAs, somebody other than yourself if you are an MP, to help you with building the vision of health equity and how you connect the dots back to be relevant for quality and safety and risk and all of those other things”
Include key stakeholders and leaders across organization	“[The office] is very much aligned because it was created by senior leadership. It was created in conjunction with senior leadership. And as we were creating the office, very early on, we aligned with the chief experience officer, the chief nursing officer, the newly created chief culture officer position, HR, and the Office of Faculty Development. So in the creation of the office, we needed such that we would be aligned with how successful offices are aligned here. It was intentional.”
Form advisory board	“One of the key leverage points for our office was the creation of our senior advisory council—senior advisory board, that’s what we called it … we said, ‘Well, we need to create a senior advisory board because every other office seems to have one. And who do we choose for the board?’ And that was the leverage point. We chose many department chiefs and chairs to be on our senior advisory board. We chose SVPs, we chose directors, we chose from the quality office, from the faculty development office, we chose medical students, … we thought we should have a student voice. We chose someone from the general council office who was—they were on an internship. We wanted to have cross-enterprise representation, but we also wanted to have potential key stakeholders. And I say potential because the office hadn’t yet really been around that long, but we knew we needed to get a board. And we got a board together and decided to have a board meeting. It was one of the most wonderful meetings because we went around the table and we asked all the board members, ‘Say why you think you’re on this board, or what you think you’d like to contribute to the office.’ And we got to hear what they think we should be doing as an Office of Health Equity and Inclusion…there were a lot of wonderful ideas around the table, but we also got to see the level of sincere commitment to seeing this office grow at such an early stage.”
Include evaluation from the beginning	“I think that there are certain types of work that we’re doing where we’re fairly advanced. And when I think about the work that’s been done over the last eight years or so in the hospital, there’s been a lot that’s been achieved. And yet, when I look at things with respect to sustainability of the work, we haven’t been able to establish concrete structures, a concrete budget, concrete staffing, concrete processes that actually would support this work.”
Develop marketing and publicity efforts	“I think that equity, generally, and diversity is on the radar for our executives. And it’s, I think, getting attention in the media. It’s recognized as being important. It’s just that hospitals haven’t done much with it. And so I think that—I don’t know if the position necessarily would help, but I think I certainly need to be doing more to profile the work we have done and profile the work that needs to get done so that we can begin to create the kind of urgency that we spoke about earlier.”
Commit “hard” money	“I think when dollars come from the Ministry, then basically, the work is being embedded in the must-do work instead of the nice-to-have work, right? So my hope and desire is to actually get funding through the global budget, the Ministry of Health, so that this work is recognized as being important work that must happen instead of it only happens if you can find funding for it. That’s the switch that we have to make.”
Develop external alliances with community and credible organizations	“[O]ur local health authority in Toronto mandated that all hospitals reporting into it that receive funding from it have to collect sociodemographic data from all of its patients and families. So things like race and ethnicity and language and sexual orientation and gender and information about disability, information about income. And so we did some incredible work, and we actually developed a pediatric health equity instrument to collect that data and implemented it. But that sense of urgency only came because it was mandated and a requirement from externally instead of something that was perhaps recognized as being really important internally in simple to provide quality care.”
Develop executive and administrative champions of the office	“So I think the administrative champions, for me, are particularly valuable at the executive level. So the vice-president, senior vice-presidents, the chiefs of nursing, the chief of pediatrics, the chief of surgery. I think if you can get folks like that or the CEO engaged and supporting the work that you’re doing, I think that that can have a lot of value. And I would say that over the last eight years or so, I’ve seen different champions come and go, but having eight champions at the table makes the difference.”
Plan for short- and long-term “wins”	“I think staying relevant to whatever the strategic priorities are has been the key. I think that also having key leaders, specifically key senior leaders that influence strategy, understand the work that we do, and understand how it fits and can be woven into the goals of the organization. I feel like I’ve needed to and will continue to connect the dots for people. No matter what we do, where we go, what goals we have, there’s always a connection back to health equity.”
Begin with narrow scope and expand over time (double-edge sword)	“It’s easy to get kind of caught in your little silo and working, but it’s really important to always be aware of who are the key people that can help you move this forward.”

## Results

Eight organizations completed the questionnaires and the in-depth interviews. All organizations are pediatric hospitals—seven are in the United States and one is in Canada. At the time of interviewing, all but two organizations had a health equity office founded by physicians and senior leadership, and all reported up to organizational leadership in the C-suite (Table 1). The scope of work varied significantly across institutions from addressing social determinants of health to DEI education to clinical quality improvement and research.

The results of our participant interviews on what is needed to organize a successful equity office are presented in Table 2 with four major themes: the history of the office is important and can determine scope of work and sphere of influence; a health equity office reduces silos and provides cross-cutting organizational direction on the execution of equity efforts; leadership buy-in is key for sustainability; and the health equity office as a central hub for data collection and analysis. Theme 5 summarizes 12 additional key elements for planning and organizing a health equity office.

## Discussion

PHEC participants articulated several key implementation factors in the founding and development of a health equity office. First, the history of the office is important and has the potential to determine the office’s scope of work and sphere of influence. Second, a health equity office can provide cross-cutting organizational direction, stability, and execution of equity efforts, reducing the effects of siloing. Third, high-level leadership buy-in provides time and financial resources that are essential for success. Finally, a health equity office should be centrally involved in the collection, analysis, and reporting of equity-focused metrics. Health equity offices in clinical settings should be involved in clinically important initiatives, services, and interventions.

All participants noted that health equity efforts are an organizational journey, and where the efforts begin determines the trajectory. For example, participants reported that where the health equity efforts were initially located in an organizational history and organizational chart determined many of the possibilities and opportunities open to a health equity office. For example, an office that started in human resources (HR) has a different mandate and limited future opportunities for growth given the focus on hiring and training. An office that starts embedded within the clinical enterprise in quality improvement and patient safety is positioned to make immediate changes on clinical services and patient outcomes. For example, Children’s Mercy Kansas City’s office began with a focus on language barriers and interpreter services access and was not initially part of a department. This allowed this office to be expanded and absorbed into Patient Care Services with a reporting structure directly to the Chief Operations Officer (COO).

Participants noted that the journey metaphor allowed for emphasizing the need for runway and landing time in starting a health equity office. Everyone noted the large amount of work and resources needed to begin and establish a health equity office, especially if an organization is new to the equity mission. A key component of creating a sustainable health equity organizational structure is time, experimentation, and an eye for organizational culture. There is not a one-size-fits-all process or a solution for health equity, but enough time and resources can lead to short-term health equity wins.^4^

It is important to note that not all participants in PHEC reported having a centralized health equity office, and some went through many years and iterations to arrive at their current health equity office. Regardless of whether or not respondents had an existing office, participants uniformly stated that having a centralized equity-focused office was essential. Having a central organized effort was seen as an important statement about the organization’s commitment to equity through dedicated financial and personnel resources. This is consistent with the organizational change management by Kotter model which emphasized the need for a having a guiding team to lead a change initiative.^4^ In addition, recent research has demonstrated that there are at least five stages of organizational DEI maturity.^8^ Initially, organizations need to become aware of their equity needs and set a collective vision for the organization. This begins the process for an intentional approach to health equity that is tailored to the unique characteristics and goals of an organization. This takes time and energy to seek multistakeholder perspectives and input. PHEC participants noted that it was important to include both internal and external to the organization stakeholders in this process. It allows for the development of relationships and partnerships that can expand the organization’s equity mission and vision.

A health equity office can help in organizing and leading the many stakeholder’s needs and demands. Office leadership can take all of these inputs and help integrate them into an organization’s mission/vision and values. PHEC participants noted that the complexity of equity efforts can result into organizations that have many efforts scattered throughout the organization, creating a diffused, decentralized, and un-orchestrated set of initiatives. This is likely to occur in most large organizations which typically have siloed and hierarchical organizational structures. For example, health equity efforts focused in HR may not be connected with clinical operations, nor with quality and outcome measurement and reporting.

One interviewee stated, “I really do not think that D&I and equity should be separated. So, in some organizations—right?—D&I is kind of tied at the hip with HR. And others, the quality equity efforts are kind of tied at the hip in quality and safety. And so they’re so closely related that I think doing that misses a picture. So, I have resisted several times when senior leaders have said, ‘Well, shouldn’t we sort of separate them? There’s a lot going on in each one of them,’ which I agree, but I think if we separate them, we’re going to really miss out on how they’re tied together.”

An office brings the possibility of health equity integration across an organization. An important DEI developmental stage is integration (stage four out of five).^8^ There is alignment of internal efforts that are both top-down and bottom-up initiatives. Moving beyond siloed health equity work allows for an overarching equity strategy, development of an equity culture and organizational structures/programming/metrics that both sustain and promulgate new experiments that are specific to an organization for recruitment and hiring, new equity clinical initiatives, and continuous monitoring and measurement of equity goals and outcomes. An overarching health equity mission and vision are essential in initiating, developing, and sustaining equity effort. Large organizations or health care systems must focus on several levels: macro-institution level vision/mission, funding and goal setting from the highest leadership levels, and operationalizing change at the local level of the organization with accountability.

PHEC participants all reported that one of the key elements to successful health equity efforts is leadership buy-in. One stated, “So, I think the administrative champions, for me, are particularly valuable at the executive level. So, the vice-president, senior vice-presidents, the chiefs of nursing, the chief of pediatrics, the chief of surgery. I think if you can get folks like that or the CEO engaged and supporting the work that you’re doing, I think that that can have a lot of value. And I would say that over the last eight years or so, I’ve seen different champions come and go, but having eight champions at the table makes the difference.” This allows for integration of equity into the vision, mission, and purpose of the organization as opposed to a secondary afterthought. This is a key component of theories of organizational change management. The Kotter model posits that the key early step for organizational change includes creating a sense of urgency and importance for health equity initiatives.^4^ The CEO and C-suite leadership have the power to champion diversity by setting the organizational agenda, setting goals, and establishing metrics and timelines in order to create accountability.^10^ It is this leadership that can lead to structural changes that can result in sustainable culture change. Sustainability is a final stage of DEI organizational development. This is when DEI initiatives and culture survive and continue to thrive despite organizational economic difficulties and changes in leadership.^8^ PHEC participants emphasized that C-suite leadership without structural changes makes health equity efforts vulnerable to leadership changes. Health equity offices can play an integral role in providing stability to organizational culture change and efforts as leadership personnel and priorities shift.

All participants stated that health equity efforts, and especially a dedicated office, should be grounded in the collection and reporting of equity-focused metrics. Metrics provide accountability and opportunities for celebration and improvement.^27^ They also create a sense of purpose and momentum. Metrics that illustrate equity issues help overcome resistance by getting buy-in for new efforts. When used to show progress, they allow additional resources to be leveraged. All participants stated that the metrics needed to be aligned with the organizational mission/vision and values and that these metrics should be developed in conjunction with the C-suite leadership. Participants also reported the importance of using metrics to create short-and long-term wins for the health equity office.

An equity-focused office provides many strengths and opportunities, but there are also challenges. Given the multiple stakeholders and competing demands, it is challenging to keep lines of communication and accountability clear and open. While the office can be the central hub, it would be difficult for an office to be responsible for and execute on all strategies. This is where the integration across the system is key, and collaboration with key stakeholders to operationalize the equity strategies. A large and complex structure may hinder implementation at the “enterprise versus local” level, therefore making collective goal setting and clear communication between the enterprise and local level, key ingredients to success. Health equity work is susceptible to changes in C-suite leadership, which necessitates the need for ensuring C-suite leadership in the health equity office, as well as structural changes within the organization to align with the equity mission. In a siloed organization, there is likely to be a diffusion of focus, for which a key ingredient to success is a collective vision for the organization. Lastly, the current environment of health care—the impact of the pandemic, financial and capacity challenges, rising costs, and staff shortage and burnout—means health equity work cannot operate in its own silo and requires strategic linkages to hospital efforts on these issues in order to align with institutional priorities and future.

In recent years, the COVID-19 pandemic and the murder of Mr. George Floyd accelerated a nationwide reckoning on structural racism, bias, and the impact on health disparities. While this certainly resulted in an increase in leadership positions focused on diversity, equity, and inclusion and a surging interest in this work,^9,28^ the current environment of health care remains a constrained one. Aligning what is needed for complete organizational transformation with the relatively limited resources and support provided to accomplish this^4,9^ makes this work still challenging. In addition, the competing demands of rising financial costs, staffing shortages and burnout, and access and capacity issues make the uptake of the building blocks for an equity office difficult.

Our study has a few limitations. Our sample is focused on a small number of large pediatric hospitals and thus our findings may not be generalizable to other settings. Our participants were sampled before the pandemic, the murder of Mr. George Floyd, and recent national discussions on equity, diversity, and inclusion. This study will provide important comparator data for future health equity studies. Health equity work is an ongoing and is a continuous process of growth and change for an organization. It will require sustained investment and continued monitoring and improvement to ensure equity is advanced. A health equity-focused office can play an integral and sustaining role in representing and focusing equity efforts across an organization, measuring processes and outcomes, and helping to continuously develop the organization’s mission and vision.

## Supplementary Material

Supplementary Appendix SA1

Supplementary Appendix SA2

Supplementary Appendix SA3

## Abbreviations Used

CEO: Chief Executive Officer
CMO: Chief Medical Officer
CNO: Chief Diversity Officer
COO: Chief Operating Officer
DEI: Diversity, Equity and Inclusion
DI: Diversity and Inclusion
DLP: Disparities Leadership Program
FTE: Full Time Equivalent
HCD: Human Centered Design
HR: Human Resources
PHEC: Pediatric Health Equity Collaborative
REL: Race, Ethinicity and Language
SVP: Senior Vice President
UCB: Unconscious Bias
UKMC: University of Missouri - Kansas City
VCH: Vanderbilt Children’s Hospital
