# Supplementary material for: Establishing a Health Equity Office: The Importance of Recentering Equity
Source: Health Equity. 2024 Aug 20;8(1):538–53. doi: 10.1089/heq.2024.0004 (PMC11347870; doi:10.1089/heq.2024.0004)
Supplement: Supplementary Appendix SA1 [file heq.2024.0004_appendix1_survey.pdf]

Appendix Table 2

PHEC Qualitative Survey Summary Questions

Domain 1: History of the Office

1. When did your office start? If possible, please provide a date.
2. What was the impetus for it starting (the why)?
3. How was the mission/vision/directive of the office created?
4. Was the creation of the office mandated?
5. Who was instrumental to creating/starting the office?

Domain 2: General Description of the Office

1. Has the mission and vision statement changed over time?
2. How has your office changed its mission/vision over time?
3. Does your office have specific directive?
4. How has your office's specific directive changed over time?
5. What is the scope of work of the office and the types of programs the office engages in? Please have a list of programs and organize your programs into big buckets: trainees, pipeline, community engagement, quality measurement, retention, etc.
6. How many personnel does your office have?
7. What type of personnel does your office have?
8. Have the personnel changed over time?
9. How have the personnel changed over time?
10. Who are the core office members and what are their titles in the office within the organization?
11. Please select the training/background of the personnel in your office. Please select all that apply.
12. Who is the leader(s) of the office?
13. How has the leadership of the office changed over time? Why has it changed?
14. Do you have affiliate members, i.e., members that are not primary to the office but are involved closely with the office?
15. How do you incorporate students or other trainees?

Domain 3: Position of the Office in the Organization

1. Where does the office sit in the organizational chart?

2. If you would like to provide a document to aid in understanding your office's organizational chart, please upload it here.
3. How has the organizational structure changed over time?
4. To whom does the office report to in the organization chart?
5. Has the reporting structure changed?
6. How has the reporting structure changed?
7. How has the reporting structure impacted the work of the Equity office? Positively? Negatively? This can be discussed further in the interviews, but a response should be provided here.
8. Are there partnership(s) with other stakeholders in the organization? e.g. HR, Academic Diversity Offices, Experience Offices.
9. Does the office have an advisory board or other external partners/advisors?

Domain 4: Budget

1. How does the office mission/vision scope correlate with allotted budget and number of personnel?
2. How do you secure a budget for FTEs for the initial Center?
3. How do you secure a budget for a growth plan(s)?
4. Do you have or plan to apply for research grants?
5. What types of grants do you have or intend to apply for?

Domain 5: Stakeholders

1. Who are your offices' stakeholders (i.e., university, medical school, community)?
2. After listing your key stakeholders, please state why these are the stakeholders that are important to the office.
3. Please describe the relationship of the stakeholders to the functioning of the office. For example, do they provide funds, personnel, place reporting requirements of the office, etc.?
4. When you have listed your stakeholders, which are active stakeholders that are closely involved in the functioning and/or funding of the office?
5. With which stakeholders are there more distant or passive (not active) relationships?

Domain 6: Community Engagement

1. Is your office involved in community engagement?
2. Please describe the type of community engagement your office is involved in.

Domain 7: Measuring Outcomes

1. How often is the office required to report outcomes or progress report(s)?
2. How are the outcomes chosen?
3. Who decides on the outcomes to report?
4. Do you produce an annual report?
5. To whom do you report annually?
6. Do you report Success stories/success?
7. How do you report success stories? In what media(s)?
8. Do you report using other media?
