# Supplementary material for: Establishing a Health Equity Office: The Importance of Recentering Equity
Source: Health Equity. 2024 Aug 20;8(1):538–53. doi: 10.1089/heq.2024.0004 (PMC11347870; doi:10.1089/heq.2024.0004)
Supplement: Supplementary Appendix SA2 [file heq.2024.0004_appendix2_interview_guide.pdf]

Appendix Table 1

PHEC Interview Guide

**Key Informant Interview Domains**
1. What is your organization's overall 'meta-stage' of development (early, middle, advanced)?<br>2. What is your current Kotter model stage now? You can describe your journey here.<br>3. How have you been able to create urgency?<br>4. How have you been able to sustain momentum/buy-in within your organization over time?<br>5. How does the office align with organizational culture?<br>6. Does your org chart location help or hinder the office? Are there better or worse org structures that you would recommend to others to use or avoid when starting an office?<br>7. Please discuss your reporting structure for your office. Is this advantageous or a hinderance? Has it changed over time? What would be the optimal reporting structure?<br>8. Please describe the funding structure for your office. What would be the optimal revenue structure for your office?<br>9. Have you been able to leverage the DLP network? The PHEC network? In what way(s)?<br>10. Does your staff oversee implementation of cross-cultural training at your organization? Please describe your efforts.<br>11. Lessons learned – advice to others on what to do and what to avoid. Are there key lessons/leverage points you used to start the office?<br>12. Reflecting on the survey results, are there longitudinal components to the development of the office that you would want to emphasize (i.e., things that you think were key to

your success?) Mention the survey domains that have changed over time in a good and/or bad way.

13. What would you do differently if you could?
14. What is the future of the office in the next year? In 5-10 years?
15. Is there someone else at your institution we should talk to?
