# Supplementary material for: Establishing a Health Equity Office: The Importance of Recentering Equity
Source: Health Equity. 2024 Aug 20;8(1):538–53. doi: 10.1089/heq.2024.0004 (PMC11347870; doi:10.1089/heq.2024.0004)
Supplement: Supplementary Appendix SA3 [file heq.2024.0004_appendix3_attendees.pdf]

Appendix 3

Attendees of the 2018 PHEC meeting at Nationwide Children's Hospital in Columbus Ohio

Boston's Children's Hospital (Nicole Tennermann, Valerie L. Ward, and Rachelle Pierre), Children's Mercy Kansas City (John Cowden, Gabriela Flores, and Marshaun Butler), Cincinnati Children's Hospital Medical Center (Aniyah Land), Johns Hopkins Medicine (Lisa Ross DeCamp), Monroe Carell Jr. Children's Hospital at Vanderbilt (Arie Nettles and Amita Bey), Nationwide Children's Hospital (LaVone Caldwell, Jane Goleman, and Olivia Thomas), Nemours/Alfred I. DuPont Hospital For Children (Kirk Dabney and Patricia Oceanic), St. Christopher's Hospital for Children (Hans Kersten), and The Hospital for Sick Children Toronto, Canada (Karima Karmali).
